# Impact of High Hydrostatic Pressure on the Physicochemical Characteristics, Functional Properties, Structure, and Bioactivities of *Tenebrio molitor* Protein

**DOI:** 10.1002/fsn3.70055

**Published:** 2025-02-18

**Authors:** Can Zhang, Huayi Suo, Jiajia Song

**Affiliations:** ^1^ College of Food Science Southwest University Chongqing People's Republic of China

**Keywords:** functional properties, high hydrostatic pressure, insect protein, non‐thermal processing technology

## Abstract

This study aimed to explore the influence of high hydrostatic pressure (HHP) treatment on the structure, functional characteristics, and bioactivities of *Tenebrio molitor* protein. The results showed that HHP induced dissociation of *T. molitor* protein, exposing hydrophobic groups and reducing particle size, which in turn reduced turbidity. Additionally, 600 MPa treatment significantly reduced the foaming stability and emulsifying activity of *T. molitor* protein. Treatments at 200 MPa and 400 MPa significantly reduced emulsion stability, whereas 400 MPa treatment significantly increased oil retention. HHP treatment also altered the secondary and tertiary structures of *T. molitor* protein, as demonstrated by circular dichroism and fluorescence spectra. Furthermore, HHP treatment significantly affected the antibacterial and antioxidant activities of *T. molitor* protein. This study provides a theoretical framework for using HHP to modify *T. molitor* protein.

## Graphical abstract

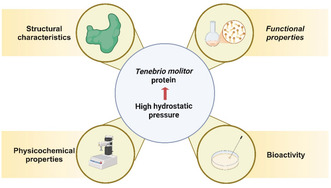

## 1 Introduction

Protein demand is increasing due to the rising global population, necessitating the identification of sustainable green protein sources. Edible insects are regarded as promising alternatives to conventional protein sources because of their high protein content, along with their economic and environmental advantages (Mannozzi et al. 2023). *Tenebrio molitor* , rich in protein and with an amino acid composition that meets human dietary needs, represents a promising insect protein resource for consumption (Pan et al. 2022). Notably, dried *T. molitor* is the first insect‐based food approved by the European Union as a novel food resource (Gonzalez‐de la Rosa et al. 2024). Current studies have reported the incorporation of *T. molitor* in various foods, including tofu, biscuits, and egg yolk‐free mayonnaise‐type products (Cozmuta et al. 2023; Gkinali et al. 2024; Oh and Kim 2024). However, consumer acceptance of edible insects remains low, limiting the widespread use of *T. molitor* in the market. Therefore, developing *T. molitor* protein products is crucial for opening a new protein market and alleviating the increasing protein demand.

Protein characteristics can be modified through various methods, such as heat treatment, ultrasound, and high hydrostatic pressure (HHP) techniques (Dehnad et al. 2024; Shen et al. 2022; Zhu et al. 2018). HHP is an innovative non‐thermal treatment technology that can inactivate pathogens and spoilage microorganisms while better preserving nutritional and sensory properties (Queirós et al. 2018). The influence of HHP treatment on protein properties has garnered significant attention. Guo et al. (2024) investigated the impact of HHP on the digestive properties and antioxidant activity of lactoferrin, demonstrating that HHP promotes lactoferrin hydrolysis without significantly affecting its antioxidant activity. Yang et al. (2024) studied the effect of HHP on the structure and allergenicity of ovalbumin, showing that HHP treatment (600 MPa) alters the secondary and tertiary structures of ovalbumin and reduces its allergenicity. Zhang et al. (2022) explored the influence of HHP on the structural and physicochemical characteristics of millet gliadin, finding that HHP increased the ordered structure, enhanced stability, and reduced hydrophobicity of millet gliadin. However, studies on the modification of *T. molitor* protein by HHP are limited, with only one study focusing on the modification of water‐soluble proteins of *T. molitor* using HHP (Boukil et al. 2022). The impact of HHP on the structural properties, functional characteristics, and bioactivity of the insoluble protein from *T. molitor* have not yet been determined.

This study explored the impact of different HHP treatments (200, 400, and 600 MPa) on the characteristics of *T. molitor* protein prepared by alkaline solubilization and acid precipitation methods. First, physicochemical characteristics, including particle size, zeta potential, micromorphology, free sulfhydryl (SH) groups, and turbidity, were analyzed using various measurement techniques. Second, functional characteristics such as solubility, foaming, emulsification, and water and oil holding capacities were examined. Additionally, structural properties were analyzed using sodium dodecyl sulfate–polyacrylamide gel electrophoresis (SDS‐PAGE), circular dichroism (CD) spectroscopy, and fluorescence spectroscopy. Finally, biological activities, including antioxidant and antibacterial activities, were evaluated. This study provides the first insights into the impact of HHP on the physicochemical, functional, and structural characteristics, as well as the biological activities of *T. molitor* insoluble protein, offering new perspectives for processing and applying *T. molitor* protein.

## 2 Materials and Methods

### 2.1 Materials

Vacuum freeze‐dried *T. molitor* powder was generously provided by Prof. Li Ning from the College of Environment and Resources, Chongqing Technology and Business University. The preformed gels used for SDS‐PAGE were obtained from Solarbio Biotech Co. Ltd. (Beijing, China). Ellman's reagent was sourced from Shanghai Macklin Biochemical Co. (Shanghai, China). All other reagents were of analytical grade.

### 2.2 Preparation of *T. molitor* Protein

The protein extraction process was carried out with slight modifications to a previously reported method (Gkinali et al. 2022). *T. molitor* was defatted using hexane. The defatted *T. molitor* was dispersed in distilled water at a ratio of 1:15 (w/v), and the pH of the solution was adjusted to 10.0. The mixture was stirred for 1 h. The supernatant was collected by centrifugation at 5000 × *g* for 20 min at 4°C. The pH of the supernatant was adjusted to 4.4 ± 0.1 and incubated for 1 h to induce protein precipitation. The precipitate was collected by centrifugation at 5000 × *g* for 20 min at 4°C and was subsequently re‐dissolved in distilled water. The pH of the solution was adjusted to 7.0. The protein solution was then dialyzed using a molecular weight cut‐off membrane of 1000 Da for 24 h at 4°C. Finally, the dialysate was freeze‐dried, yielding a protein content of 85.2% ± 0.9%, as determined by the Kjeldahl method.

### 2.3 HHP Treatment

*T. molitor* protein underwent HHP treatment using an HHP apparatus (SHPP‐2 L, Leadford Technology Co., Shanxi, China). The protein was dispersed in distilled water at a concentration of 2% (w/v). The pH of the suspension was adjusted to 9.0 and stirred for 2 h. After degassing, the solution was sealed in a polyethylene bag and subjected to HHP treatments at 200, 400, and 600 MPa for 10 min (Huang et al. 2023). The treated protein solutions were vacuum freeze‐dried and stored at 4°C.

### 2.4 Determination of Physicochemical Characteristics of *T. molitor* Protein After HHP Treatment

#### 2.4.1 Particle Size

A laser particle sizer (Mastersizer 3000; Malvern Instruments Co. Ltd., Malvern, UK) was used to determine the particle size distribution curve and volume mean diameter (*D*_*43*_). The refractive indices for the particles and dispersant were set to 1.48 and 1.33, respectively. The samples were diluted directly in the measuring cell of the instrument to achieve 5% masking (Boukil et al. 2022; Tan et al. 2021).

#### 2.4.2 Zeta Potential

Sample solutions were made at a concentration of 2 mg/mL, and the zeta potential was measured using a Zetasizer (Nano ZS90; Malvern Instruments Co. Ltd., Malvern, UK) (Kang et al. 2023).

#### 2.4.3 Scanning Electron Microscope

Samples were gold‐sputtered under vacuum, and images were obtained at 10 kV accelerating voltage using scanning electron microscope (SEM; Phenom Pro, Phenom World, Eindhoven, Netherlands) (Zhang et al. 2022).

#### 2.4.4 Free SH Content

Surface SH concentration was analyzed using Ellman's reagent as described previously (Tan et al. 2021). Sample solutions (2 mg/mL) were prepared in 0.086 mol/L Tris‐glycine buffer at pH 8.0, centrifuged at 10,000 × *g* for 20 min, and the supernatant was then collected. Ellman's reagent (4 mg/mL) was added to the supernatant at 1% (v/v) and incubated in the dark for 15 min. Optical density at 412 nm was measured. SH concentration was calculated using the following equation:

$$\mathrm{SH}\;\left(\text{μmol}/g\right)=\frac{73.53\times A\times D}{C}$$

where *A* is the optical density, *D* is the dilution factor, and *C* is the sample concentration (mg/mL).

#### 2.4.5 Turbidity

Turbidity was measured as previously described with minor modifications (Huang et al. 2023). Sample solutions (1.5 mg/mL) were prepared and vortexed to homogeneity. Optical density at 660 nm was measured using a microplate reader (Synergy H1; BioTek Instruments Inc., Winooski, VT, USA).

### 2.5 Determination of Functional Properties of *T. molitor* Protein After HHP Treatment

#### 2.5.1 Solubility

The sample was dispersed in deionized water to a final concentration of 1% (w/v), centrifuged at 8000 × *g* for 15 min, and the supernatant was collected (Zhang et al. 2023). Protein content in the supernatant was determined using the Bradford method. Solubility was calculated using the equation:

$$\text{Solubility}\;\left(\%\right)=\frac{m_{0}}{m_{1}}\times 100$$

where *m*_0_ is the protein content of the supernatant and *m*_1_ is the protein content of the suspension.

#### 2.5.2 Foaming Capacity and Foaming Stability

Foaming capacity (FC) and foaming stability (FS) were determined as previously described (Huang et al. 2023). A 40 mL sample solution (0.5% w/v) was prepared, and its initial volume (*V*_1_) was recorded. The solution was then homogenized at 10,000 rpm for 3 min, and the volume of the foam (*V*_2_) and after 30 min of standing (*V*_3_) were recorded. FC and FS were calculated using the following equations:

$$\mathrm{FC}\;\left(\%\right)=\frac{V_{2}-V_{1}}{V_{1}}\times 100$$

$$\mathrm{FS}\;\left(\%\right)=\frac{V_{3}-V_{1}}{V_{1}}\times 100$$

#### 2.5.3 Emulsion Activity Index (EAI) and Emulsion Stability Index (ESI)

Emulsion activity index (EAI) and emulsion stability index (ESI) were determined as previously described (Jiang et al. 2021). A 30 mL sample solution (0.5%) was mixed with 10 mL of soybean oil and homogenized for 5 min at 10,000 rpm. 50 μL of the emulsion was sampled from the bottom at 0 and 10 min and added to 5 mL of 0.1% (w/v) sodium dodecyl sulfate solution. Optical density at 500 nm was measured. EAI and ESI were calculated as follows:

$$\mathrm{EAI}\;\left(m^{2}/g\right)=\frac{2\times 2.303\times A_{0}\times \mathrm{DF}}{C\times \emptyset \times 1000}$$

$$\mathrm{ESI}\;\left(\min\right)=\frac{A_{0}}{∆A}\times 10$$

where DF is the dilution factor (100), ∅ is the volume fraction of oil (0.25), *C* is the sample concentration (mg/mL), and ∆A is the difference between *A*_0_ and *A*_10_.

#### 2.5.4 Water Holding Capacity and Oil Holding Capacity

1 g of sample was mixed with 20 mL of distilled water or soybean oil, mixed well, and centrifuged at 8000 × *g* for 10 min. The residue was weighed, and the results were expressed as grams of water/oil absorbed per gram of sample (Bing et al. 2024).

### 2.6 Determination of Protein Structure of *T. molitor* After HHP Treatment

#### 2.6.1 SDS‐PAGE

A sample solution (10 mg/mL) was prepared and mixed with an equal volume of loading buffer (Beyotime Biotechnology Co. Ltd., Jiangsu, China), then boiled for 5 min. After cooling to room temperature, 10 μL of the mixed solution from each sample was loaded into separate wells. Electrophoresis was conducted at a constant voltage of 120 V for 90 min. The gel was stained for 1 h with Coomassie Brilliant Blue R‐250 (0.25%) and de‐stained for 12 h using a solution containing 10% methanol and 10% acetic acid.

#### 2.6.2 CD Analysis

The secondary structure of the samples was analyzed using CD (J‐810; Jasco International Co. Ltd., Tokyo, Japan) as described previously (Zhao et al. 2022). Samples were prepared to a concentration of 0.10 mg/mL, scanned over a wavelength range of 190–250 nm, with three parallel measurements. The analysis was performed using the BeStSel website (https://bestsel.elte.hu/index.php).

#### 2.6.3 Intrinsic Fluorescence Spectroscopy

The fluorescence intensity of the sample solution (2 mg/mL) was measured using a fluorescence spectrophotometer (F‐4700; Hitachi Ltd., Tokyo, Japan) as described previously with minor modifications (Liu et al. 2023). Fluorescence spectra were scanned in the range of 310–580 nm at an excitation wavelength of 295 nm (slit width = 5.0 nm).

#### 2.6.4 Surface Hydrophobicity

Surface hydrophobicity was assessed as previously reported with minor modifications (Kang et al. 2023). Sample solutions were prepared at concentrations of 0.1–0.5 mg/mL. 1‐Anilino‐8‐naphthalene‐sulfonate (8 mmol/L) was added to the sample solution at 0.5% (v/v). After 10 min of reaction, fluorescence intensity was measured under an excitation wavelength of 390 nm and an emission wavelength of 470 nm. Surface hydrophobicity was determined from the slope of the linear regression of the fluorescence intensity and protein concentration.

### 2.7 Determination of Biological Activity of *T. molitor* Protein After HHP Treatment

#### 2.7.1 Antibacterial Activity

The 150 μL of Luria–Bertani (LB) medium, 40 μL of filtered sample solution (2 mg/mL), and 10 μL 10^6^ CFU/mL of saline pre‐diluted indicator strain ( *Staphylococcus aureus* and *Escherichia coli* ) were mixed and incubated. LB medium without strain and sample solution was used as the blank control, LB medium with strain but without the sample solution was used as the positive control, and LB medium with the sample solution but without strain was used as the negative control. OD_600_ was measured after 24 h of incubation at 37°C (Zhao et al. 2022). The antibacterial rate was calculated as follows:

$$\text{Antibacterial rate}\;\left(\%\right)=\left(1-\frac{A_{3}-A_{2}}{A_{1}-A_{0}}\right)\times 100$$

where *A*_0_, *A*_1_, *A*_2_, and *A*_3_ represent the OD_600_ values of blank control, positive control, negative control, and sample, respectively.

#### 2.7.2 2,2‐Diphenyl‐1‐Picrylhydrazyl (DPPH) Radical Scavenging Activity

An equal volume of 0.1 mmol/L 2,2‐diphenyl‐1‐picrylhydrazyl (DPPH) in anhydrous ethanol was mixed with the sample solution and incubated for 30 min away from light. Optical density was measured at 517 nm (Zhang et al. 2024). DPPH radical scavenging activity was calculated using the following formula:

$$\text{DPPH radical scavenging activity}\;\left(\%\right)=\left(1-\frac{\left(A_{2}-A_{3}\right)}{A_{1}}\right)\times 100$$

where *A*_1_, *A*_2_, and *A*_3_ represent the OD_517_ of the control, the sample with and without DPPH solution, respectively.

### 2.8 Statistical Analysis

All results were presented as mean ± standard deviation. The differences were considered significant at *p* < 0.05. One‐way analysis of variance and Tukey's test using SPSS v26.0 (SPSS Inc., Chicago, Illinois, USA) were applied to compare means.

## 3 Results and Discussion

### 3.1 Effect of HHP on Physicochemical Properties of *T. molitor* Protein

**FIGURE 1:**
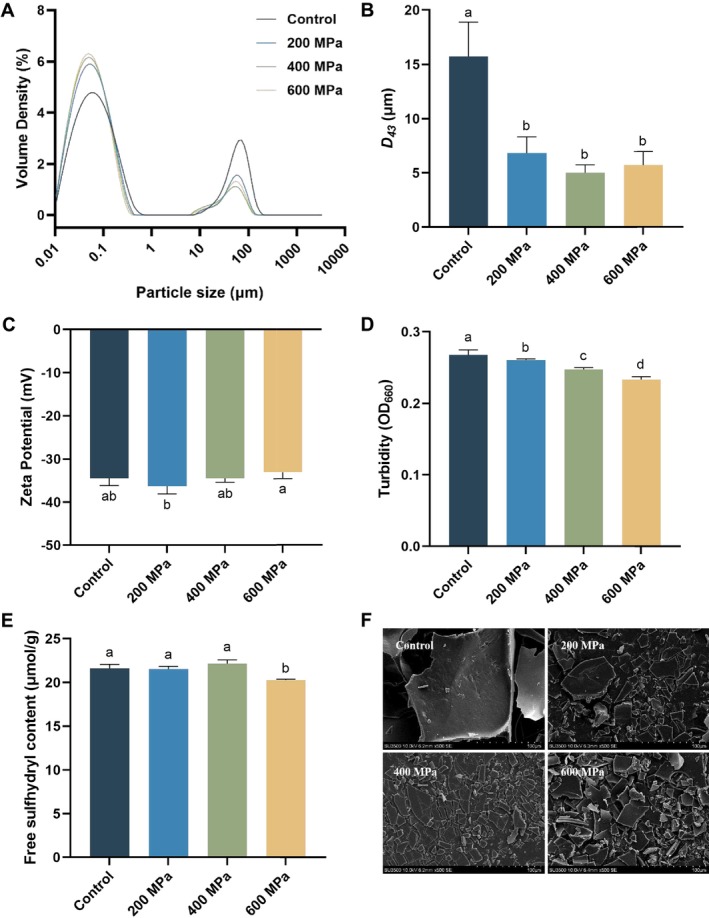
Effect of high hydrostatic pressure (HHP) on the particle size distributions (A), volume mean diameter (*D*_*43*_) (B), zeta potential (C), turbidity (D), free sulfhydryl content (E), and scanning electron microscopy (F) (500 × magnification) of control (untreated) and HHP‐treated *Tenebrio molitor* protein. Different letters represent significant differences among groups (*p* < 0.05).

Figure 1A,B illustrates the impact of HHP on the particle size of *T. molitor* protein. Figure 1A shows that the particle size distribution of *T. molitor* protein was bimodal both before and after HHP treatment. The number of small particles (0.01–0.1 μm) in the protein solution increased, whereas the number of large particles (10–100 μm) decreased after HHP treatment. Figure 1B demonstrates that the volume average diameter (D43) of *T. molitor* protein significantly decreased after HHP treatment. These findings suggest that HHP treatment reduced the particle size of *T. molitor* protein, possibly due to the dissociation of protein aggregates caused by shear force and turbulence effects (Ding et al. 2022). A similar phenomenon was observed in the study of kafirin (Yang et al. 2023).

The impact of HHP treatment on the zeta potential of *T. molitor* protein is shown in Figure 1C. The isoelectric point of *T. molitor* protein is around 4.0, and it is negatively charged when the pH of the sample solution is 9.0 (Huang et al. 2022). Compared to untreated *T. molitor* protein, the zeta potential showed no significant change after HHP treatment, likely because HHP treatment had minimal impact on the volume of *T. molitor* protein, resulting in no notable change in electrostatic interaction (Li et al. 2011). However, the absolute value of zeta potential after 600 MPa treatment was significantly lower than that after 200 MPa treatment. This difference may be associated with the partial reaggregation of *T. molitor* protein induced by the 600 MPa treatment, which reduces the number of charged amino acids on the protein surfaces (Zhao et al. 2018).

Turbidity is generally related to particle aggregation (Dehnad et al. 2023). Figure 1D illustrates that the turbidity of *T. molitor* protein significantly reduced after HHP treatment, indicating that HHP treatment dissociated *T. molitor* protein aggregates. This observation was consistent with findings from particle size distribution and D43 analysis. A similar phenomenon was observed in the study of scallop mantle myofibrillar protein (Liu et al. 2022).

The dissociation and aggregation of proteins are closely related to changes in free SH content (Wang et al. 2022). Figure 1E illustrates the impact of HHP treatment on the free SH content of *T. molitor* protein. The free SH content decreased significantly after 600 MPa treatment in comparison with the control group. The reduction in free SH content may be due to the oxidation of free SH groups to form disulfide bonds (Zhang et al. 2022). Additionally, the 600 MPa treatment may cause some *T. molitor* proteins to re‐aggregate, further decreasing the free SH content, which was consistent with previous studies by Luo et al. (2022).

The surface morphology of *T. molitor* protein treated with different high pressures was observed by SEM. Figure 1F shows the surface morphology of untreated and HHP‐treated *T. molitor* protein. Untreated *T. molitor* protein exhibited a large sheet structure with irregular edges. After 200 MPa treatment, *T. molitor* protein displayed a relatively small, loose, flaky structure, although the edges remained irregular. After 400 MPa treatment, *T. molitor* protein exhibited a smaller and more uniform size distribution, with smoother edges. However, compared to the 400 MPa group, *T. molitor* protein treated at 600 MPa showed some large, irregular flakes. This is likely due to the re‐aggregation of some proteins under 600 MPa treatment, which aligned with the particle size distribution results. Similar findings were observed in the previous studies (Ding et al. 2022; Wang et al. 2022).

### 3.2 Effect of HHP on Functional Properties of *T. molitor* Protein

**FIGURE 2:**
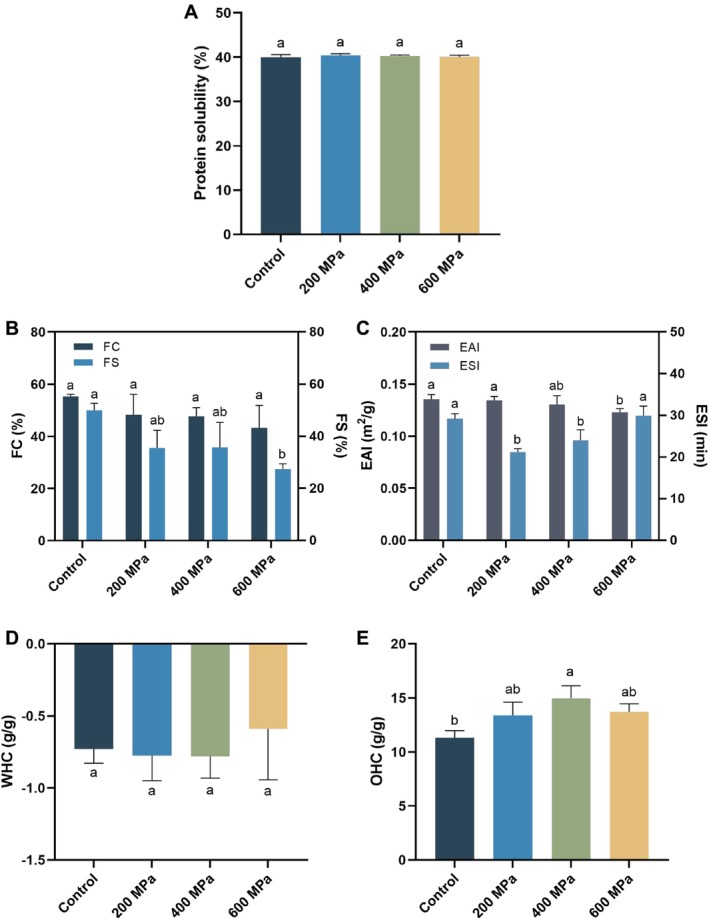
Effect of high hydrostatic pressure (HHP) on the solubility (A), emulsion activity index (EAI) and emulsion stability index (ESI) (B), foaming capacity (FC) and foaming stability (FS) (C), water holding capacity (WHC) (D), and oil holding capacity (OHC) (E) of control (untreated) and HHP‐treated *Tenebrio molitor* protein. Different letters represent significant differences among groups (*p* < 0.05).

The effect of HHP treatment on the solubility of *T. molitor* protein is shown in Figure 2A. The solubility of HHP‐treated *T. molitor* protein did not change considerably in comparison with the untreated protein. Similar findings were observed in studies of pea protein (Kalayci et al. 2023) and sweet potato protein (Zhao et al. 2018), which may be attributed to the sample solution's pH being 9.0. The impact of pH on solubility was greater than the impact of HHP on solubility.

The foaming characteristics reflect the stability of protein at the water‐air interface (Huang et al. 2024). *T. molitor* protein treated with HHP did not show a significant difference in FC compared to the untreated protein, as depicted in Figure 2B. However, FS considerably decreased with 600 MPa treatment in comparison with the control group. The mismatch between the flexibility and stiffness of the *T. molitor* protein structure at high pressure may cause the FS decline (Li et al. 2011; Wang et al. 2022).

EAI and ESI represent the emulsifying ability and stability of protein, which measure their ability to produce and stabilize oil under aqueous conditions. As shown in Figure 2C, the EAI was considerably lower after 600 MPa treatment compared to the control. This may be due to the 600 MPa treatment causing some *T. molitor* protein to polymerize, leading to the loss of proteins at the interface, consequently lowering the EAI, which was comparable to the previous study of Yin et al. (2008). Molecular flexibility has been reported to correlate with emulsion stability (Wang et al. 2022). The ESI dramatically dropped after 200 MPa and 400 MPa treatments compared to the control group, possibly because HHP treatment reduced molecular flexibility.

Water holding capacity (WHC) and oil holding capacity (OHC) measure the quantity of water or oil absorbed and retained in 1 g of protein (Bing et al. 2024). WHC was negligible and did not significantly differ, as illustrated in Figure 2D. This may be because the sample solution's pH was 9.0, at which the solubility of *T. molitor* protein was good, resulting in almost no precipitate after centrifugation. Compared with untreated *T. molitor* protein, OHC increased significantly after 400 MPa treatment, as shown in Figure 2E. The contact between oil and protein can occur when the fatty chain of oil joins with the nonpolar chain of amino acids. Therefore, the more hydrophobic a protein is, the greater its OHC (Zhang et al. 2023). The increase in OHC after 400 MPa treatment may result from hydrophobic groups being exposed.

### 3.3 Effect of HHP on Structural Characteristics of *T. molitor* Protein

**FIGURE 3:**
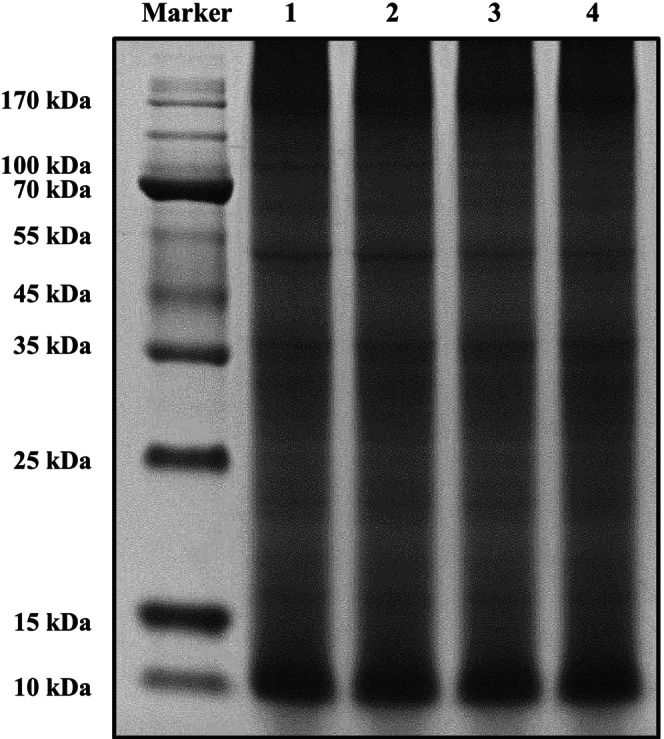
Sodium dodecyl sulfate–polyacrylamide gel electrophoresis (SDS‐PAGE) analysis of control (untreated) and high hydrostatic pressure‐treated *Tenebrio molitor* protein. Lane 1: Control; lanes 2–4: *Tenebrio molitor* protein treated with 200, 400, and 600 MPa, respectively.

Figure 3 shows the impact of HHP on the primary structure of *T. molitor* protein. The band of 8.5–13 kDa may be antifreeze protein, and the 14–32 kDa band may be stratum corneum protein (Huang et al. 2023). The 32–100 kDa band may be associated with enzymes and other relevant proteins, including melanin inhibitory protein (43 kDa), β‐glucosidase (59 kDa), and other proteins linked to melanin synthesis (85 kDa) (Gkinali et al. 2022). After HHP treatment, the distribution of *T. molitor* protein bands did not notably alter from the control group, suggesting that HHP had no discernible impact on the primary structure of *T. molitor* protein.

**FIGURE 4:**
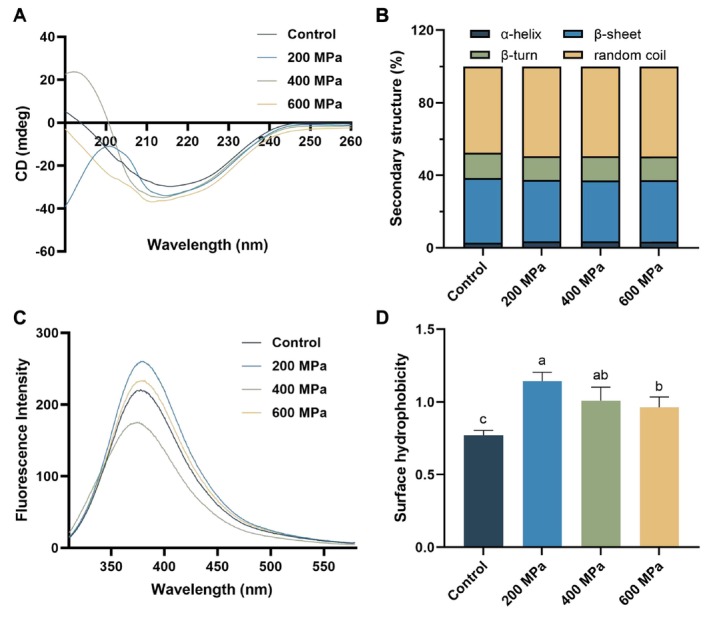
Effect of high hydrostatic pressure (HHP) on the circular dichroism (CD) spectra analysis (A), secondary structure contents (B), intrinsic fluorescence spectrum (C), and surface hydrophobicity (D) of control (untreated) and HHP‐treated *Tenebrio molitor* protein. Different letters represent significant differences among groups (*p* < 0.05).

CD spectrum analysis was used to evaluate the impact of HHP on the secondary structure of *T. molitor* protein. As illustrated in Figure 4A, HHP affected the CD spectrum intensity of *T. molitor* protein, indicating changes in the secondary structure. Figure 4B shows the secondary structure contents of *T. molitor* protein, including α‐helix, β‐sheet, β‐turn, and random coil. The findings showed that HHP increased the content of α‐helix and random coil but decreased the content of β‐sheet and β‐turn, indicating that some β‐sheets changed to α‐helices. A comparable finding was reported in the study of β‐lactoglobulin (Kieserling et al. 2021). β‐sheets depend on the hydrogen bond between peptide bonds. As the pressure generated by protein expansion increased, the hydrogen bonds were destroyed, leading to certain β‐sheets becoming α‐helices (Liu et al. 2022).

Figure 4C shows the endogenous fluorescence spectrum of *T. molitor* protein before and after HHP treatment. HHP treatment changed the fluorescence intensity and emission wavelength of *T. molitor* protein. The relative fluorescence intensity of *T. molitor* protein increased after 200 MPa and 600 MPa treatment in comparison with the control group, suggesting that protein unfolding increased the contact between tryptophan and solvent (Yu et al. 2023). However, the relative fluorescence intensity decreased after 400 MPa treatment, possibly due to a shift in the surrounding environment of tryptophan residues, resulting in some tryptophan masking (Kang et al. 2023). The change in protein excitation fluorescence emission wavelength signals a modification in the protein's molecular structure. Changes in protein excitation fluorescence emission wavelength signal modifications in the protein's molecular structure. The maximum emission wavelength (λmax) of the control group was about 377 nm. The λmax of *T. molitor* protein after 200 MPa and 600 MPa treatment was ~379 nm, showing a slight red shift compared to the control group, suggesting that the 200 MPa and 600 MPa treatments increased the polarity of the surrounding environment of tryptophan (Liu et al. 2023), likely due to the expansion of protein structure and enhanced water‐tryptophan interaction (Yu et al. 2023). Following 400 MPa treatment, the λmax of *T. molitor* protein was ~376 nm, showing a slight red shift, which may be because the contact between tryptophan and water was reduced after 400 MPa treatment.

Figure 4D illustrates the surface hydrophobicity of untreated and HHP‐treated *T. molitor* protein. After HHP treatment, the surface hydrophobicity of *T. molitor* protein considerably increased due to the expansion of protein structure and the exposure of hidden hydrophobic groups (Luo et al. 2022). However, compared with 200 MPa treatment, the surface hydrophobicity after 600 MPa treatment significantly reduced, possibly because some *T. molitor* proteins re‐aggregated after 600 MPa treatment, causing the exposed hydrophobic groups to become re‐hidden (Dehnad et al. 2023). This was consistent with the particle size distribution results.

### 3.4 Effect of HHP on Biological Activity of *T. molitor* Protein

**FIGURE 5:**
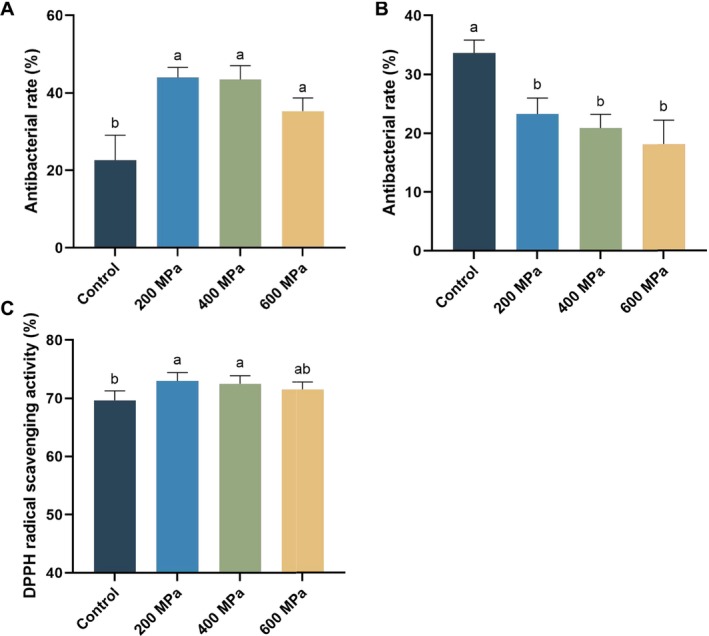
Effect of high hydrostatic pressure (HHP) on the biological activity of control (untreated) and HHP‐treated *Tenebrio molitor* protein: Antibacterial activity against *Staphylococcus aureus* (A) and *Escherichia coli* (B); 2,2‐diphenyl‐1‐picrylhydrazyl (DPPH) radical scavenging activity (C). Different letters represent significant differences among groups (*p* < 0.05).

The influence of HHP on the antibacterial activity of *T. molitor* protein is shown in Figure 5A,B. HHP treatment significantly enhanced the antibacterial rate of *T. molitor* protein against *S. aureus* (Figure 5A). This enhancement may be due to protein dissociation caused by HHP treatment, which exposed antibacterial fragments of *T. molitor* protein with growth inhibitory effects on *S. aureus* . However, high‐pressure treatment significantly reduced the antibacterial rate of *T. molitor* protein against *E. coli* (Figure 5B), similar to findings by Modugno et al. (2018). This reduction may be because HHP destroyed the protein fragments of *T. molitor* protein that had growth inhibitory effects on *E. coli* .

To evaluate the impact of HHP on the antioxidant activity of *T. molitor* protein, the DPPH free radical scavenging activity was analyzed. As shown in Figure 5C, the DPPH free radical scavenging activity after 200 MPa and 400 MPa treatment was considerably enhanced in comparison to the control group. In contrast, the DPPH free radical scavenging activity after 600 MPa treatment was slightly less than that after 200 MPa and 400 MPa treatment. It is reported that hydrophobic amino acids such as Tyr and Trp enhance the antioxidant activity of samples (Yu et al. 2023). Therefore, this result may be due to 200 MPa and 400 MPa treatments causing protein unfolding and exposure of hydrophobic groups, whereas the 600 MPa treatment caused some proteins to regroup, reducing the exposure of hydrophobic groups. This observation aligned with the tertiary structure analysis.

## 4 Conclusions

This research described the alterations to *T. molitor* protein characteristics observed under HHP treatment. The primary structure of *T. molitor* protein was not considerably altered by HHP treatment, but the secondary and tertiary structures were affected, resulting in increased surface hydrophobicity. In addition, HHP reduced the particle size of *T. molitor* protein, as confirmed by SEM analysis. The reduction in particle size led to a decrease in turbidity. Notably, 600 MPa treatment significantly reduced the amount of free SH groups. HHP treatment also altered the functional characteristics of *T. molitor* protein, including emulsification, foaming, and oil retention. Furthermore, HHP treatment modified the antibacterial and antioxidant activities of *T. molitor* protein. In summary, HHP treatment may benefit the processing of *T. molitor* protein, providing new insights for its application in the food industry.

## Author Contributions

**Can Zhang:** data curation (equal), investigation (equal), methodology (equal), visualization (equal), writing – original draft (equal). **Huayi Suo:** writing – review and editing (equal). **Jiajia Song:** conceptualization (equal), funding acquisition (equal), project administration (equal), supervision (equal), writing – review and editing (equal).

## Conflicts of Interest

The authors declare no conflicts of interest.

## Data Availability

The data that support the findings of this study are available from the corresponding author upon reasonable request.
